# *catena*-Poly[[[aqua­(4,4′-dimethyl-2,2′-bipyridine-κ^2^ *N*,*N*′)zinc]-μ-3-chloro­benzene-1,2-dicaboxylato-κ^2^ *O* ^2^:*O* ^3^] [[(4,4′-dimethyl-2,2′-bipyridine-κ^2^ *N*,*N*′)zinc]-μ-3-chloro­benzene-1,2-dicaboxylato-κ^2^ *O* ^2^:*O* ^3^]]

**DOI:** 10.1107/S1600536812014006

**Published:** 2012-04-13

**Authors:** Yu Zhu, Hai-Yang Li, Ming-Li Ma

**Affiliations:** a Department of Chemistry, Zhengzhou University, Zhengzhou 450001, People’s Republic of China

## Abstract

In the title compound, {[Zn(C_8_H_3_ClO_4_)(C_12_H_12_N_2_)(H_2_O)]·[Zn(C_8_H_3_ClO_4_)(C_12_H_12_N_2_)]}_*n*_, one Zn^2+^ ion is five-coordin­ated by two O atoms from two different 3-chloro­benzene-1,2-dicarboxyl­ate ligands, one O atom from a water mol­ecule and two N atoms from a 4,4′-dimethyl-2,2′-bipyridine ligand, while the second Zn^2+^ ion is four-coordinated by two O atoms from two different 3-chloro­benzene-1,2-dicarboxyl­ate ligands, and two N atoms from a 4,4′-bimethyl-2,2′-bipyridine ligand. The crystal structure exhibits a three-dimensional supra­molecular structure composed of alternate Zn(C_8_H_3_O_4_Cl)(C_12_H_12_N_2_) and Zn(C_8_H_3_O_4_Cl)(C_12_H_12_N_2_)(H_2_O) chains, which are linked together by face-to-face π–π inter­actions [shortest centroid–centroid distances of 3.661 (4) and 3.6901 (3) Å], O—H⋯O and C—H⋯O hydrogen bonds.

## Related literature

For background to the network topologies and applications of coordination polymers, see: Maspoch *et al.* (2007 ▶); Ockwig *et al.* (2005 ▶); Zang *et al.* (2011 ▶). For related O—H⋯O hydrogen bonds, see: Desiraju *et al.* (2004 ▶). For related π–π inter­actions, see: Zang *et al.* (2010 ▶). For related C—H⋯O hydrogen bonds, see: Desiraju *et al.* (1996 ▶). For related C—H⋯π inter­actions, see: Nishio *et al.* (1998 ▶).
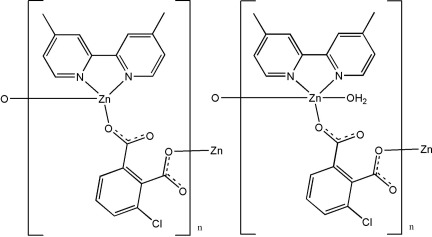


## Experimental

### 

#### Crystal data

[Zn(C_8_H_3_ClO_4_)(C_12_H_12_N_2_)(H_2_O)]·[Zn(C_8_H_3_ClO_4_)(C_12_H_12_N_2_)]
*M* *_r_* = 914.38
Orthorhombic, 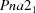

*a* = 34.050 (4) Å
*b* = 14.1831 (10) Å
*c* = 7.8764 (6) Å
*V* = 3803.8 (6) Å^3^
*Z* = 4
Mo *K*α radiation
μ = 1.47 mm^−1^
*T* = 291 K
0.20 × 0.18 × 0.16 mm

#### Data collection

Oxford Diffraction Xcalibur Eos Gemini diffractometer
Absorption correction: multi-scan (*CrysAlis PRO*; Oxford Diffraction, 2007 ▶) *T*_min_ = 0.971, *T*_max_ = 1.000
10533 measured reflections
6396 independent reflections
5076 reflections with *I* > 2σ(*I*)
*R*_int_ = 0.044

#### Refinement

*R*[*F* ^2^ > 2σ(*F* ^2^)] = 0.056
*wR*(*F* ^2^) = 0.104
*S* = 1.07
6396 reflections
518 parameters
1 restraint
H-atom parameters constrained
Δρ_max_ = 0.63 e Å^−3^
Δρ_min_ = −0.37 e Å^−3^
Absolute structure: Flack (1983 ▶), 2200 Friedel pairs
Flack parameter: 0.093 (14)

### 

Data collection: *CrysAlis PRO* (Oxford Diffraction, 2007 ▶); cell refinement: *CrysAlis PRO*; data reduction: *CrysAlis PRO*; program(s) used to solve structure: *SHELXS97* (Sheldrick, 2008 ▶); program(s) used to refine structure: *SHELXL97* (Sheldrick, 2008 ▶); molecular graphics: *OLEX2* (Dolomanov *et al.*, 2009 ▶); software used to prepare material for publication: *OLEX2*.

## Supplementary Material

Crystal structure: contains datablock(s) I, global. DOI: 10.1107/S1600536812014006/zq2156sup1.cif

Structure factors: contains datablock(s) I. DOI: 10.1107/S1600536812014006/zq2156Isup2.hkl

Additional supplementary materials:  crystallographic information; 3D view; checkCIF report

## Figures and Tables

**Table 1:** Hydrogen-bond geometry (Å, °)

*D*—H⋯*A*	*D*—H	H⋯*A*	*D*⋯*A*	*D*—H⋯*A*
O1*W*—H1*WA*⋯O4^i^	0.85	2.10	2.768 (6)	135
O1*W*—H1*WB*⋯O4^ii^	0.85	1.79	2.644 (6)	179
O1*W*—H1*WB*⋯O3^ii^	0.85	2.43	2.930 (5)	118
C17—H17⋯O4^i^	0.93	2.36	3.202 (8)	150
C20—H20⋯O6^iii^	0.93	2.28	3.171 (8)	160
C23—H23⋯O6^iii^	0.93	2.40	3.306 (8)	164
C32—H32⋯O2^i^	0.93	2.57	3.459 (8)	161
C35—H35⋯O2^i^	0.93	2.45	3.353 (8)	165

Symmetry codes: (i) 
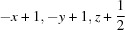
; (ii) 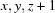; (iii) 
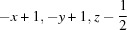
.

**Fig. 1.**
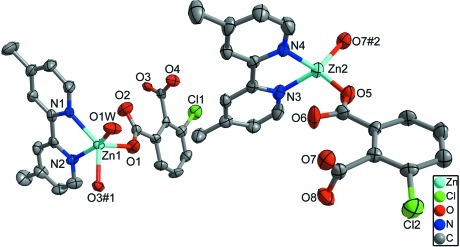
Metal coordination and atom labeling in the title compound (thermal ellipsoids drawn at the 50% probability level). All hydrogen atoms are omitted for clarity. Symmetry code: x, y, z - 1.

**Fig. 2.**
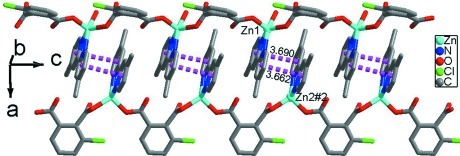
A view of the layered structure of the title compound. Dotted lines represent the π–π interactions (symmetry code: -x + 1, -y + 1, z + 0.5.)

**Fig. 3.**
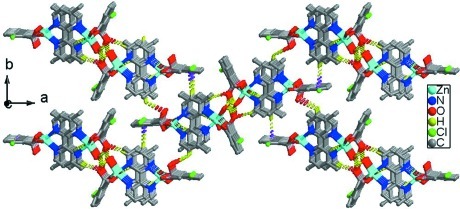
The three-dimensional supramolecular structure of the title compound (hydrogen bonds and C—H–π interactions as dotted lines).

## supplementary crystallographic information

### Comment

In recent years, metallosupramolecular compounds have received much attention due to their variety of architectures and the potential applications as functional materials (Maspoch *et al.*, 2007; Ockwig *et al.*, 2005). Early reports have shown that carboxylate compounds and nitrogen heterocyclic ligands have been successfully employed in the generation of many novel structures (Zang *et al.*, 2011). To further explore various factors that influence the properties and construction of coordination compounds, we undertake synthetic and structural studies on a novel Zn^II^ complex based on 3-chlorophthalic acid (H2cp) and 4,4'-dimethyl-2,2'-bipyridine (dmbpy).

The X-ray diffraction study revealed that the title compound crystallizes in the orthorhombic space group *Pna*2_1_. The Zn1 atom is five-coordinated by two O atoms from two different 3-chlorobenzene-1,2-dicarboxylato ligands, one O atom from a water molecule and two N atoms from a 4,4'-dimethyl-2,2'-bipyridine ligand, while the Zn2 atom is four-coordinated by two O atoms from two different 3-chlorobenzene-1,2-dicarboxylato ligands, and two N atoms from a 4,4'-bimethyl-2,2'-bipyridine ligand (Fig. 1). Each cp^2-^ ligand acts as a *µ*_2_-bridge linking two Zn atoms with both carboxylate groups in monodentate fashion resulting into two chains along the *c* axis. These two chains are further connected together by face-to-face π–π interactions (Zang *et al.*, 2010) involving pyridine rings of different chains. The shortest *Cg*···*Cg* distances of 3.661 (4) and 3.6901 (3) are observed between *Cg*2 and *Cg*4, and *Cg*1 and *Cg*3, respectively (*Cg*1 is the centroid of the N1/C17–C21 ring, *Cg*2 of N2/C22–C26, *Cg*3 of N3/C34–C38, and *Cg*4 of N4/C29–C33). Adjacent zippered structures are linked together to form tetra-chain units by intermolecular O1w—H1wa···O4*^iii^* hydrogen bonds (Desiraju *et al.*, 2004) and C17—H17···O4*^iii^* interactions (symmetry code: *iii* = -*x* + 1, -*y* + 1, *z* + 1/2). Each unit is further connected to other units through C20—H20···O6*^iv^*, C23—H23···O6*^iv^* (symmetry code: *iv* = -*x* + 1, -*y* + 1, *z* - 1/2), C32—H32···O2*^iii^*, C35—H35···O2*^iii^* (Desiraju *et al.*, 1996) and C—H···π interactions (the shortest one being C37—H37···*Cg*5*^v^* = 2.79 Å; *Cg*5 centroid of C11–C16; symmetry code: *v* = -*x* + 3/2, *y* - 1/2, *z* - 1/2) (Nishio *et al.*, 1998) to form a three-dimensional supramolecular structure (Fig. 3).

### Experimental

The title compound was synthesized hydrothermally in a Teflon-lined stainless steel container by heating a mixture of 3-chlorophthalic acid (H_2_cp) (0.0100 g, 0.05 mmol), 4,4'-bimethyl-2,2'-bipyridine (dmbpy) (0.0092 g, 0.05 mmol), Zn(NO_3_)_2_.6H_2_O (0.0149 g, 0.05 mmol) and NaOH (0.0040 g, 0.1 mmol) in 7 ml of distilled water at 120°C for 3 days, and then cooled to room temperature. Colourless block crystals were obtained in 71% yield (based on zinc).

### Refinement

H atoms were positioned geometrically and refined using a riding model, with C—H = 0.93 Å for aromatic H atoms, C—H = 0.97 Å for methylene H atoms [with *U*_iso_(H) = 1.2*U*_eq_(C)], and O—H = 0.85 Å for the water H atoms [with *U*_iso_(H) = 1.5*U*_eq_(O)].

### Crystal data

**Table:**

[Zn(C_8_H_3_ClO_4_)(C_12_H_12_N_2_)(H_2_O)]·[Zn(C_8_H_3_ClO_4_)(C_12_H_12_N_2_)]	*D*_x_ = 1.597 Mg m^−^^3^
*M**_r_* = 914.38	Mo *K*α radiation, λ = 0.7107 Å
Orthorhombic, *P**n**a*2_1_	Cell parameters from 1842 reflections
*a* = 34.050 (4) Å	θ = 2.9–29.3°
*b* = 14.1831 (10) Å	µ = 1.47 mm^−^^1^
*c* = 7.8764 (6) Å	*T* = 291 K
*V* = 3803.8 (6) Å^3^	Prismatic, colourless
*Z* = 4	0.20 × 0.18 × 0.16 mm
*F*(000) = 1864	

### Data collection

**Table:**

Oxford Diffraction Xcalibur Eos Gemini diffractometer	6396 independent reflections
Radiation source: Enhance (Mo) X-ray Source	5076 reflections with *I* > 2σ(*I*)
Graphite monochromator	*R*_int_ = 0.044
Detector resolution: 16.2312 pixels mm^-1^	θ_max_ = 26.4°, θ_min_ = 2.9°
ω scans	*h* = −18→42
Absorption correction: multi-scan (*CrysAlis PRO*; Oxford Diffraction, 2007)	*k* = −17→17
*T*_min_ = 0.971, *T*_max_ = 1.000	*l* = −7→9
10533 measured reflections	

### Refinement

**Table:**

Refinement on *F*^2^	Secondary atom site location: difference Fourier map
Least-squares matrix: full	Hydrogen site location: inferred from neighbouring sites
*R*[*F*^2^ > 2σ(*F*^2^)] = 0.056	H-atom parameters constrained
*wR*(*F*^2^) = 0.104	*w* = 1/[σ^2^(*F*_o_^2^) + (0.0355*P*)^2^] where *P* = (*F*_o_^2^ + 2*F*_c_^2^)/3
*S* = 1.07	(Δ/σ)_max_ = 0.001
6396 reflections	Δρ_max_ = 0.63 e Å^−^^3^
518 parameters	Δρ_min_ = −0.37 e Å^−^^3^
1 restraint	Absolute structure: Flack (1983), 2200 Friedel pairs
Primary atom site location: structure-invariant direct methods	Flack parameter: 0.093 (14)

### Special details

**Table:**

Geometry. All e.s.d.'s (except the e.s.d. in the dihedral angle between two l.s. planes) are estimated using the full covariance matrix. The cell e.s.d.'s are taken into account individually in the estimation of e.s.d.'s in distances, angles and torsion angles; correlations between e.s.d.'s in cell parameters are only used when they are defined by crystal symmetry. An approximate (isotropic) treatment of cell e.s.d.'s is used for estimating e.s.d.'s involving l.s. planes.
Refinement. Refinement of *F*^2^ against ALL reflections. The weighted *R*-factor *wR* and goodness of fit *S* are based on *F*^2^, conventional *R*-factors *R* are based on *F*, with *F* set to zero for negative *F*^2^. The threshold expression of *F*^2^ > σ(*F*^2^) is used only for calculating *R*-factors(gt) *etc*. and is not relevant to the choice of reflections for refinement. *R*-factors based on *F*^2^ are statistically about twice as large as those based on *F*, and *R*-factors based on ALL data will be even larger.

### Fractional atomic coordinates and isotropic or equivalent isotropic displacement parameters (Å^2^)

**Table:**

	*x*	*y*	*z*	*U*_iso_*/*U*_eq_	
Zn1	0.446526 (19)	0.36444 (4)	0.62772 (9)	0.02863 (16)	
Zn2	0.73030 (2)	0.67276 (5)	0.37248 (10)	0.03441 (18)	
Cl1	0.53270 (7)	0.15962 (15)	−0.2478 (2)	0.0655 (6)	
Cl2	0.84880 (6)	0.65033 (13)	1.2682 (2)	0.0487 (5)	
O1	0.48307 (15)	0.3004 (3)	0.4755 (5)	0.0462 (13)	
O1W	0.47741 (14)	0.4839 (3)	0.7036 (6)	0.0493 (13)	
H1WA	0.4918	0.5028	0.6220	0.074*	
H1WB	0.4881	0.4524	0.7830	0.074*	
O2	0.45488 (16)	0.3521 (3)	0.2380 (6)	0.0524 (14)	
O3	0.46114 (11)	0.2980 (3)	−0.1501 (5)	0.0289 (9)	
O4	0.51092 (14)	0.3849 (3)	−0.0516 (5)	0.0450 (12)	
O5	0.77114 (14)	0.6635 (4)	0.5400 (6)	0.0487 (13)	
O6	0.73636 (14)	0.6683 (4)	0.7724 (6)	0.0651 (17)	
O7	0.75751 (13)	0.7136 (3)	1.1661 (5)	0.0400 (11)	
O8	0.76227 (13)	0.5605 (3)	1.1177 (7)	0.0553 (13)	
N1	0.39925 (14)	0.4462 (3)	0.5397 (6)	0.0282 (12)	
N2	0.39966 (14)	0.2639 (3)	0.6065 (7)	0.0315 (12)	
N3	0.68490 (14)	0.5810 (3)	0.3955 (6)	0.0335 (12)	
N4	0.68816 (15)	0.7651 (3)	0.4601 (6)	0.0329 (13)	
C1	0.47656 (19)	0.2992 (5)	0.3164 (7)	0.0305 (15)	
C1A	0.49032 (19)	0.3121 (4)	−0.0579 (7)	0.0266 (14)	
C2	0.5919 (2)	0.9246 (5)	0.6602 (12)	0.072 (3)	
H2A	0.5960	0.9384	0.7781	0.107*	
H2B	0.5682	0.8888	0.6473	0.107*	
H2C	0.5898	0.9824	0.5975	0.107*	
C3	0.49776 (19)	0.2223 (4)	0.2198 (7)	0.0264 (14)	
C4	0.50368 (18)	0.2278 (4)	0.0461 (7)	0.0263 (14)	
C5	0.5226 (2)	0.1537 (4)	−0.0331 (9)	0.0388 (18)	
C6	0.5346 (2)	0.0737 (5)	0.0544 (9)	0.0463 (19)	
H6	0.5470	0.0246	−0.0028	0.056*	
C7	0.5282 (2)	0.0680 (5)	0.2251 (9)	0.0447 (19)	
H7	0.5356	0.0143	0.2846	0.054*	
C8	0.5105 (2)	0.1420 (4)	0.3089 (8)	0.0404 (18)	
H8	0.5071	0.1388	0.4258	0.049*	
C9	0.7673 (2)	0.6666 (5)	0.7003 (9)	0.0356 (17)	
C10	0.77225 (18)	0.6434 (4)	1.0906 (7)	0.0325 (15)	
C11	0.80563 (19)	0.6689 (4)	0.7983 (7)	0.0292 (15)	
C12	0.80732 (19)	0.6604 (4)	0.9751 (7)	0.0251 (14)	
C13	0.8441 (2)	0.6620 (4)	1.0484 (8)	0.0308 (15)	
C14	0.8786 (2)	0.6732 (4)	0.9570 (9)	0.0384 (17)	
H14	0.9028	0.6751	1.0115	0.046*	
C15	0.8759 (2)	0.6812 (5)	0.7839 (10)	0.0473 (19)	
H15	0.8986	0.6874	0.7192	0.057*	
C16	0.8403 (2)	0.6802 (4)	0.7065 (7)	0.0354 (16)	
H16	0.8390	0.6873	0.5892	0.043*	
C17	0.4015 (2)	0.5397 (4)	0.5072 (9)	0.0426 (17)	
H17	0.4248	0.5717	0.5298	0.051*	
C18	0.3702 (2)	0.5882 (4)	0.4417 (9)	0.0448 (18)	
H18	0.3724	0.6526	0.4209	0.054*	
C19	0.3356 (2)	0.5428 (4)	0.4066 (8)	0.0404 (17)	
C20	0.33365 (19)	0.4466 (4)	0.4388 (8)	0.0378 (16)	
H20	0.3105	0.4136	0.4181	0.045*	
C21	0.36621 (18)	0.4002 (4)	0.5020 (7)	0.0281 (14)	
C22	0.36610 (18)	0.2978 (4)	0.5409 (7)	0.0285 (14)	
C23	0.33407 (19)	0.2400 (4)	0.5094 (8)	0.0371 (16)	
H23	0.3112	0.2650	0.4631	0.045*	
C24	0.3363 (2)	0.1447 (4)	0.5475 (9)	0.0441 (18)	
C25	0.3715 (2)	0.1119 (4)	0.6178 (11)	0.0478 (18)	
H25	0.3743	0.0485	0.6453	0.057*	
C26	0.4011 (2)	0.1728 (4)	0.6454 (9)	0.0471 (18)	
H26	0.4240	0.1497	0.6947	0.057*	
C27	0.3001 (2)	0.5942 (5)	0.3372 (12)	0.074 (3)	
H27A	0.2881	0.6302	0.4263	0.111*	
H27B	0.2816	0.5492	0.2937	0.111*	
H27C	0.3082	0.6357	0.2475	0.111*	
C28	0.3008 (2)	0.0812 (5)	0.5202 (10)	0.064 (2)	
H28A	0.2998	0.0345	0.6084	0.096*	
H28B	0.3029	0.0506	0.4119	0.096*	
H28C	0.2772	0.1184	0.5233	0.096*	
C29	0.6922 (2)	0.8562 (5)	0.4932 (10)	0.0499 (19)	
H29	0.7161	0.8853	0.4702	0.060*	
C30	0.6618 (2)	0.9098 (4)	0.5612 (9)	0.049 (2)	
H30	0.6656	0.9734	0.5847	0.059*	
C31	0.62615 (19)	0.8681 (4)	0.5935 (9)	0.0410 (18)	
C32	0.62257 (19)	0.7715 (4)	0.5612 (8)	0.0356 (16)	
H32	0.5989	0.7408	0.5827	0.043*	
C33	0.65364 (19)	0.7224 (4)	0.4983 (7)	0.0290 (14)	
C34	0.65222 (19)	0.6193 (4)	0.4647 (7)	0.0296 (15)	
C35	0.61938 (19)	0.5652 (4)	0.5012 (7)	0.0351 (16)	
H35	0.5973	0.5938	0.5480	0.042*	
C36	0.6189 (2)	0.4691 (4)	0.4691 (8)	0.0384 (17)	
C37	0.6532 (2)	0.4317 (4)	0.3981 (10)	0.0473 (19)	
H37	0.6547	0.3676	0.3745	0.057*	
C38	0.6845 (2)	0.4887 (4)	0.3632 (10)	0.0460 (17)	
H38	0.7067	0.4617	0.3144	0.055*	
C39	0.5834 (2)	0.4107 (5)	0.5081 (10)	0.059 (2)	
H39A	0.5900	0.3642	0.5920	0.088*	
H39B	0.5746	0.3799	0.4066	0.088*	
H39C	0.5629	0.4506	0.5509	0.088*	

### Atomic displacement parameters (Å^2^)

**Table:**

	*U*^11^	*U*^22^	*U*^33^	*U*^12^	*U*^13^	*U*^23^
Zn1	0.0277 (4)	0.0328 (3)	0.0254 (3)	0.0020 (3)	0.0000 (4)	0.0016 (3)
Zn2	0.0264 (4)	0.0442 (4)	0.0327 (4)	0.0009 (3)	0.0010 (4)	−0.0032 (4)
Cl1	0.0765 (16)	0.0915 (15)	0.0283 (10)	0.0279 (13)	0.0089 (10)	−0.0091 (10)
Cl2	0.0505 (13)	0.0658 (12)	0.0297 (9)	0.0041 (10)	−0.0054 (8)	−0.0054 (8)
O1	0.053 (3)	0.070 (3)	0.016 (2)	0.021 (3)	0.002 (2)	−0.005 (2)
O1W	0.060 (4)	0.040 (3)	0.048 (3)	−0.014 (2)	−0.011 (2)	0.015 (2)
O2	0.071 (4)	0.056 (3)	0.031 (3)	0.027 (3)	−0.005 (3)	−0.011 (2)
O3	0.029 (2)	0.038 (2)	0.020 (2)	−0.0047 (18)	−0.0028 (19)	0.0053 (19)
O4	0.048 (3)	0.044 (3)	0.043 (3)	−0.015 (2)	−0.017 (2)	0.007 (2)
O5	0.034 (3)	0.081 (4)	0.030 (3)	0.004 (3)	−0.003 (2)	−0.001 (2)
O6	0.025 (3)	0.127 (5)	0.043 (3)	−0.006 (3)	0.003 (3)	0.007 (3)
O7	0.041 (3)	0.048 (3)	0.032 (3)	0.008 (2)	0.004 (2)	−0.010 (2)
O8	0.048 (3)	0.041 (3)	0.077 (3)	−0.002 (2)	0.018 (3)	0.015 (3)
N1	0.024 (3)	0.027 (3)	0.033 (3)	0.001 (2)	0.008 (2)	0.000 (2)
N2	0.032 (3)	0.029 (3)	0.033 (3)	−0.001 (2)	−0.006 (3)	0.001 (2)
N3	0.031 (3)	0.033 (3)	0.037 (3)	0.003 (2)	0.000 (3)	−0.011 (3)
N4	0.024 (3)	0.030 (3)	0.045 (3)	0.001 (2)	0.000 (2)	−0.007 (2)
C1	0.025 (4)	0.039 (4)	0.028 (3)	−0.001 (3)	0.002 (3)	0.000 (3)
C1A	0.028 (4)	0.033 (3)	0.019 (3)	−0.002 (3)	0.002 (3)	−0.004 (3)
C2	0.050 (5)	0.046 (4)	0.120 (8)	−0.003 (4)	0.028 (5)	−0.024 (5)
C3	0.029 (4)	0.032 (3)	0.019 (3)	−0.002 (3)	−0.005 (3)	−0.003 (3)
C4	0.017 (3)	0.032 (3)	0.029 (3)	−0.003 (3)	−0.004 (3)	−0.004 (3)
C5	0.038 (5)	0.037 (4)	0.041 (4)	−0.001 (3)	−0.007 (3)	−0.004 (3)
C6	0.048 (5)	0.037 (4)	0.054 (5)	0.011 (4)	−0.003 (4)	−0.007 (3)
C7	0.057 (5)	0.039 (4)	0.038 (4)	0.013 (4)	−0.015 (4)	0.007 (3)
C8	0.049 (5)	0.044 (4)	0.027 (4)	0.000 (4)	−0.007 (3)	0.004 (3)
C9	0.035 (4)	0.040 (4)	0.033 (4)	0.005 (3)	0.008 (3)	0.002 (3)
C10	0.030 (4)	0.045 (4)	0.022 (4)	−0.003 (3)	−0.004 (3)	−0.003 (3)
C11	0.027 (4)	0.029 (3)	0.031 (3)	0.003 (3)	−0.002 (3)	−0.004 (3)
C12	0.029 (4)	0.023 (3)	0.023 (3)	0.000 (3)	0.000 (3)	−0.006 (2)
C13	0.039 (4)	0.025 (3)	0.028 (3)	0.005 (3)	0.000 (3)	−0.002 (3)
C14	0.034 (4)	0.048 (4)	0.033 (4)	−0.003 (3)	0.001 (3)	−0.005 (3)
C15	0.028 (4)	0.059 (5)	0.055 (5)	0.002 (4)	0.007 (4)	0.000 (4)
C16	0.043 (5)	0.049 (4)	0.014 (3)	−0.002 (3)	−0.001 (3)	−0.002 (3)
C17	0.033 (4)	0.030 (3)	0.064 (5)	0.003 (3)	0.009 (4)	0.004 (3)
C18	0.042 (5)	0.026 (3)	0.066 (5)	0.008 (3)	0.007 (4)	0.001 (3)
C19	0.036 (4)	0.042 (4)	0.043 (4)	0.012 (3)	0.000 (3)	0.000 (3)
C20	0.027 (4)	0.042 (4)	0.044 (4)	0.002 (3)	−0.002 (3)	0.008 (3)
C21	0.022 (3)	0.038 (3)	0.024 (3)	−0.003 (3)	0.002 (3)	−0.005 (3)
C22	0.028 (4)	0.041 (3)	0.017 (3)	0.000 (3)	0.002 (3)	−0.002 (3)
C23	0.026 (4)	0.038 (4)	0.047 (4)	−0.005 (3)	−0.001 (3)	−0.005 (3)
C24	0.051 (5)	0.035 (4)	0.047 (4)	−0.015 (4)	−0.001 (4)	−0.005 (3)
C25	0.064 (5)	0.025 (3)	0.055 (4)	0.002 (3)	−0.006 (5)	0.001 (4)
C26	0.054 (5)	0.039 (3)	0.048 (4)	−0.003 (3)	−0.014 (4)	0.005 (4)
C27	0.053 (5)	0.057 (5)	0.112 (8)	0.018 (4)	−0.018 (6)	0.022 (5)
C28	0.059 (6)	0.048 (4)	0.086 (6)	−0.023 (4)	−0.006 (5)	−0.005 (4)
C29	0.040 (5)	0.046 (4)	0.064 (5)	−0.011 (4)	0.011 (4)	−0.004 (4)
C30	0.043 (4)	0.028 (3)	0.076 (5)	−0.005 (3)	0.003 (4)	−0.011 (3)
C31	0.034 (4)	0.040 (3)	0.050 (5)	0.008 (3)	0.006 (3)	−0.015 (3)
C32	0.028 (4)	0.038 (4)	0.041 (4)	−0.005 (3)	0.001 (3)	−0.004 (3)
C33	0.030 (4)	0.031 (3)	0.026 (3)	0.000 (3)	−0.006 (3)	−0.002 (3)
C34	0.027 (4)	0.034 (3)	0.027 (3)	0.000 (3)	−0.002 (3)	−0.004 (3)
C35	0.025 (4)	0.043 (4)	0.038 (4)	−0.004 (3)	0.000 (3)	0.000 (3)
C36	0.039 (4)	0.031 (3)	0.045 (4)	−0.004 (3)	−0.011 (3)	−0.001 (3)
C37	0.047 (5)	0.029 (3)	0.066 (5)	−0.001 (3)	0.002 (4)	−0.002 (4)
C38	0.034 (4)	0.043 (4)	0.061 (5)	0.007 (3)	−0.001 (4)	−0.012 (4)
C39	0.044 (5)	0.034 (4)	0.098 (6)	0.000 (4)	−0.001 (5)	0.007 (4)

### Geometric parameters (Å, º)

**Table:**

Zn1—O1	1.952 (4)	C12—C13	1.378 (9)
Zn1—O1W	2.082 (4)	C13—C14	1.387 (9)
Zn1—O3^i^	2.049 (4)	C14—H14	0.9300
Zn1—N1	2.102 (5)	C14—C15	1.371 (8)
Zn1—N2	2.146 (4)	C15—H15	0.9300
Zn2—O5	1.921 (5)	C15—C16	1.357 (9)
Zn2—O7^ii^	1.958 (4)	C16—H16	0.9300
Zn2—N3	2.029 (5)	C17—H17	0.9300
Zn2—N4	2.062 (5)	C17—C18	1.370 (9)
Cl1—C5	1.729 (7)	C18—H18	0.9300
Cl2—C13	1.747 (6)	C18—C19	1.372 (9)
O1—C1	1.273 (7)	C19—C20	1.389 (8)
O1W—H1WA	0.8512	C19—C27	1.513 (9)
O1W—H1WB	0.8501	C20—H20	0.9300
O2—C1	1.220 (8)	C20—C21	1.382 (8)
O3—Zn1^ii^	2.049 (4)	C21—C22	1.484 (8)
O3—C1A	1.246 (7)	C22—C23	1.387 (8)
O4—C1A	1.249 (7)	C23—H23	0.9300
O5—C9	1.270 (7)	C23—C24	1.386 (9)
O6—C9	1.196 (8)	C24—C25	1.400 (9)
O7—Zn2^i^	1.958 (4)	C24—C28	1.523 (9)
O7—C10	1.264 (7)	C25—H25	0.9300
O8—C10	1.242 (7)	C25—C26	1.347 (8)
N1—C17	1.352 (8)	C26—H26	0.9300
N1—C21	1.334 (7)	C27—H27A	0.9600
N2—C22	1.343 (7)	C27—H27B	0.9600
N2—C26	1.329 (7)	C27—H27C	0.9600
N3—C34	1.353 (7)	C28—H28A	0.9600
N3—C38	1.334 (7)	C28—H28B	0.9600
N4—C29	1.325 (8)	C28—H28C	0.9600
N4—C33	1.357 (8)	C29—H29	0.9300
C1—C3	1.514 (8)	C29—C30	1.391 (9)
C1A—C4	1.520 (8)	C30—H30	0.9300
C2—H2A	0.9600	C30—C31	1.374 (9)
C2—H2B	0.9600	C31—C32	1.400 (8)
C2—H2C	0.9600	C32—H32	0.9300
C2—C31	1.507 (8)	C32—C33	1.360 (8)
C3—C4	1.385 (7)	C33—C34	1.487 (8)
C3—C8	1.407 (8)	C34—C35	1.386 (8)
C4—C5	1.381 (9)	C35—H35	0.9300
C5—C6	1.389 (9)	C35—C36	1.386 (8)
C6—H6	0.9300	C36—C37	1.399 (9)
C6—C7	1.365 (9)	C36—C39	1.498 (9)
C7—H7	0.9300	C37—H37	0.9300
C7—C8	1.377 (9)	C37—C38	1.364 (9)
C8—H8	0.9300	C38—H38	0.9300
C9—C11	1.518 (9)	C39—H39A	0.9600
C10—C12	1.521 (9)	C39—H39B	0.9600
C11—C12	1.399 (7)	C39—H39C	0.9600
C11—C16	1.394 (9)		
			
O1—Zn1—O1W	103.5 (2)	C16—C15—C14	120.3 (7)
O1—Zn1—O3^i^	98.96 (17)	C16—C15—H15	119.9
O1—Zn1—N1	122.84 (18)	C11—C16—H16	119.2
O1—Zn1—N2	96.7 (2)	C15—C16—C11	121.7 (6)
O1W—Zn1—N1	91.85 (18)	C15—C16—H16	119.2
O1W—Zn1—N2	159.80 (19)	N1—C17—H17	119.4
O3^i^—Zn1—O1W	90.36 (16)	N1—C17—C18	121.3 (6)
O3^i^—Zn1—N1	136.19 (17)	C18—C17—H17	119.4
O3^i^—Zn1—N2	86.66 (17)	C17—C18—H18	119.7
N1—Zn1—N2	76.79 (18)	C17—C18—C19	120.6 (6)
O5—Zn2—O7^ii^	104.35 (19)	C19—C18—H18	119.7
O5—Zn2—N3	116.5 (2)	C18—C19—C20	117.7 (6)
O5—Zn2—N4	108.5 (2)	C18—C19—C27	122.1 (6)
O7^ii^—Zn2—N3	128.6 (2)	C20—C19—C27	120.1 (6)
O7^ii^—Zn2—N4	114.78 (19)	C19—C20—H20	120.1
N3—Zn2—N4	81.22 (19)	C21—C20—C19	119.7 (6)
C1—O1—Zn1	120.0 (4)	C21—C20—H20	120.1
Zn1—O1W—H1WA	109.3	N1—C21—C20	121.6 (6)
Zn1—O1W—H1WB	89.9	N1—C21—C22	115.8 (5)
H1WA—O1W—H1WB	118.4	C20—C21—C22	122.6 (6)
C1A—O3—Zn1^ii^	128.1 (4)	N2—C22—C21	115.3 (5)
C9—O5—Zn2	127.2 (5)	N2—C22—C23	121.8 (5)
C10—O7—Zn2^i^	110.2 (4)	C23—C22—C21	122.9 (6)
C17—N1—Zn1	124.0 (4)	C22—C23—H23	120.2
C21—N1—Zn1	116.7 (4)	C24—C23—C22	119.7 (6)
C21—N1—C17	119.1 (6)	C24—C23—H23	120.2
C22—N2—Zn1	115.1 (4)	C23—C24—C25	117.1 (6)
C26—N2—Zn1	126.8 (4)	C23—C24—C28	120.2 (7)
C26—N2—C22	118.0 (5)	C25—C24—C28	122.6 (6)
C34—N3—Zn2	113.9 (4)	C24—C25—H25	120.2
C38—N3—Zn2	128.4 (4)	C26—C25—C24	119.6 (6)
C38—N3—C34	117.5 (5)	C26—C25—H25	120.2
C29—N4—Zn2	127.8 (5)	N2—C26—C25	123.9 (6)
C29—N4—C33	118.8 (6)	N2—C26—H26	118.1
C33—N4—Zn2	113.2 (4)	C25—C26—H26	118.1
O1—C1—C3	114.9 (6)	C19—C27—H27A	109.5
O2—C1—O1	126.6 (6)	C19—C27—H27B	109.5
O2—C1—C3	118.5 (5)	C19—C27—H27C	109.5
O3—C1A—O4	127.1 (6)	H27A—C27—H27B	109.5
O3—C1A—C4	115.2 (5)	H27A—C27—H27C	109.5
O4—C1A—C4	117.4 (5)	H27B—C27—H27C	109.5
H2A—C2—H2B	109.5	C24—C28—H28A	109.5
H2A—C2—H2C	109.5	C24—C28—H28B	109.5
H2B—C2—H2C	109.5	C24—C28—H28C	109.5
C31—C2—H2A	109.5	H28A—C28—H28B	109.5
C31—C2—H2B	109.5	H28A—C28—H28C	109.5
C31—C2—H2C	109.5	H28B—C28—H28C	109.5
C4—C3—C1	121.7 (6)	N4—C29—H29	118.9
C4—C3—C8	119.5 (6)	N4—C29—C30	122.1 (6)
C8—C3—C1	118.7 (5)	C30—C29—H29	118.9
C3—C4—C1A	122.2 (6)	C29—C30—H30	120.2
C5—C4—C1A	119.7 (5)	C31—C30—C29	119.6 (6)
C5—C4—C3	118.1 (6)	C31—C30—H30	120.2
C4—C5—Cl1	119.9 (5)	C30—C31—C2	121.3 (6)
C4—C5—C6	122.3 (6)	C30—C31—C32	117.7 (6)
C6—C5—Cl1	117.8 (5)	C32—C31—C2	121.1 (6)
C5—C6—H6	120.3	C31—C32—H32	120.0
C7—C6—C5	119.3 (7)	C33—C32—C31	120.0 (6)
C7—C6—H6	120.3	C33—C32—H32	120.0
C6—C7—H7	120.1	N4—C33—C32	121.7 (5)
C6—C7—C8	119.8 (6)	N4—C33—C34	115.4 (5)
C8—C7—H7	120.1	C32—C33—C34	122.9 (6)
C3—C8—H8	119.6	N3—C34—C33	116.1 (5)
C7—C8—C3	120.9 (6)	N3—C34—C35	121.7 (5)
C7—C8—H8	119.6	C35—C34—C33	122.2 (6)
O5—C9—C11	114.7 (6)	C34—C35—H35	119.5
O6—C9—O5	124.3 (7)	C36—C35—C34	121.0 (6)
O6—C9—C11	121.0 (6)	C36—C35—H35	119.5
O7—C10—C12	117.9 (5)	C35—C36—C37	115.9 (6)
O8—C10—O7	123.8 (6)	C35—C36—C39	121.0 (6)
O8—C10—C12	117.9 (6)	C37—C36—C39	123.1 (6)
C12—C11—C9	122.7 (6)	C36—C37—H37	119.8
C16—C11—C9	117.9 (6)	C38—C37—C36	120.5 (6)
C16—C11—C12	119.4 (6)	C38—C37—H37	119.8
C11—C12—C10	125.2 (6)	N3—C38—C37	123.4 (6)
C13—C12—C10	117.7 (5)	N3—C38—H38	118.3
C13—C12—C11	117.0 (6)	C37—C38—H38	118.3
C12—C13—Cl2	119.9 (5)	C36—C39—H39A	109.5
C12—C13—C14	123.6 (6)	C36—C39—H39B	109.5
C14—C13—Cl2	116.6 (5)	C36—C39—H39C	109.5
C13—C14—H14	121.0	H39A—C39—H39B	109.5
C15—C14—C13	118.0 (8)	H39A—C39—H39C	109.5
C15—C14—H14	121.0	H39B—C39—H39C	109.5
C14—C15—H15	119.9		
			
Zn1—O1—C1—O2	18.8 (10)	N3—C34—C35—C36	0.5 (9)
Zn1—O1—C1—C3	−159.9 (4)	N4—Zn2—O5—C9	34.9 (7)
Zn1^ii^—O3—C1A—O4	22.0 (9)	N4—Zn2—N3—C34	−4.7 (4)
Zn1^ii^—O3—C1A—C4	−152.6 (4)	N4—Zn2—N3—C38	−179.3 (6)
Zn1—N1—C17—C18	176.6 (5)	N4—C29—C30—C31	−1.0 (11)
Zn1—N1—C21—C20	−178.3 (5)	N4—C33—C34—N3	−1.9 (8)
Zn1—N1—C21—C22	4.9 (6)	N4—C33—C34—C35	178.3 (6)
Zn1—N2—C22—C21	−3.0 (6)	C1—C3—C4—C1A	1.6 (10)
Zn1—N2—C22—C23	175.9 (4)	C1—C3—C4—C5	−178.7 (6)
Zn1—N2—C26—C25	−175.1 (6)	C1—C3—C8—C7	176.6 (6)
Zn2—O5—C9—O6	6.5 (12)	C1A—C4—C5—Cl1	2.7 (9)
Zn2—O5—C9—C11	−173.1 (4)	C1A—C4—C5—C6	−178.4 (6)
Zn2^i^—O7—C10—O8	17.5 (8)	C2—C31—C32—C33	178.9 (7)
Zn2^i^—O7—C10—C12	−155.3 (4)	C3—C4—C5—Cl1	−177.0 (5)
Zn2—N3—C34—C33	5.0 (6)	C3—C4—C5—C6	1.9 (10)
Zn2—N3—C34—C35	−175.2 (4)	C4—C3—C8—C7	−1.3 (10)
Zn2—N3—C38—C37	173.7 (6)	C4—C5—C6—C7	−0.9 (12)
Zn2—N4—C29—C30	−176.3 (5)	C5—C6—C7—C8	−1.3 (12)
Zn2—N4—C33—C32	178.6 (5)	C6—C7—C8—C3	2.3 (11)
Zn2—N4—C33—C34	−2.0 (6)	C8—C3—C4—C1A	179.5 (6)
Cl1—C5—C6—C7	178.0 (6)	C8—C3—C4—C5	−0.8 (10)
Cl2—C13—C14—C15	179.1 (5)	C9—C11—C12—C10	3.1 (9)
O1—Zn1—N1—C17	−90.0 (5)	C9—C11—C12—C13	178.9 (5)
O1—Zn1—N1—C21	84.6 (4)	C9—C11—C16—C15	−178.7 (6)
O1—Zn1—N2—C22	−118.1 (4)	C10—C12—C13—Cl2	−3.1 (7)
O1—Zn1—N2—C26	59.4 (6)	C10—C12—C13—C14	177.4 (6)
O1—C1—C3—C4	−160.0 (6)	C11—C12—C13—Cl2	−179.2 (4)
O1—C1—C3—C8	22.1 (9)	C11—C12—C13—C14	1.3 (9)
O1W—Zn1—O1—C1	−108.3 (5)	C12—C11—C16—C15	1.5 (10)
O1W—Zn1—N1—C17	17.3 (5)	C12—C13—C14—C15	−1.4 (10)
O1W—Zn1—N1—C21	−168.0 (4)	C13—C14—C15—C16	1.4 (11)
O1W—Zn1—N2—C22	61.4 (8)	C14—C15—C16—C11	−1.5 (11)
O1W—Zn1—N2—C26	−121.2 (6)	C16—C11—C12—C10	−177.1 (6)
O2—C1—C3—C4	21.2 (10)	C16—C11—C12—C13	−1.3 (8)
O2—C1—C3—C8	−156.7 (6)	C17—N1—C21—C20	−3.4 (9)
O3^i^—Zn1—O1—C1	159.2 (5)	C17—N1—C21—C22	179.8 (5)
O3^i^—Zn1—N1—C17	109.8 (5)	C17—C18—C19—C20	−0.3 (10)
O3^i^—Zn1—N1—C21	−75.6 (5)	C17—C18—C19—C27	178.9 (7)
O3^i^—Zn1—N2—C22	143.3 (4)	C18—C19—C20—C21	−0.9 (9)
O3^i^—Zn1—N2—C26	−39.3 (6)	C19—C20—C21—N1	2.8 (9)
O3—C1A—C4—C3	−106.9 (7)	C19—C20—C21—C22	179.4 (5)
O3—C1A—C4—C5	73.4 (7)	C20—C21—C22—N2	−178.0 (6)
O4—C1A—C4—C3	77.9 (8)	C20—C21—C22—C23	3.2 (9)
O4—C1A—C4—C5	−101.8 (7)	C21—N1—C17—C18	2.1 (9)
O5—Zn2—N3—C34	101.7 (4)	C21—C22—C23—C24	179.3 (6)
O5—Zn2—N3—C38	−72.9 (6)	C22—N2—C26—C25	2.3 (11)
O5—Zn2—N4—C29	63.2 (6)	C22—C23—C24—C25	0.4 (9)
O5—Zn2—N4—C33	−111.5 (4)	C22—C23—C24—C28	177.5 (6)
O5—C9—C11—C12	−171.7 (6)	C23—C24—C25—C26	0.1 (11)
O5—C9—C11—C16	8.5 (9)	C24—C25—C26—N2	−1.4 (12)
O6—C9—C11—C12	8.7 (10)	C26—N2—C22—C21	179.3 (6)
O6—C9—C11—C16	−171.1 (7)	C26—N2—C22—C23	−1.8 (9)
O7^ii^—Zn2—O5—C9	157.7 (6)	C27—C19—C20—C21	179.9 (7)
O7^ii^—Zn2—N3—C34	−119.5 (4)	C28—C24—C25—C26	−177.0 (7)
O7^ii^—Zn2—N3—C38	65.9 (7)	C29—N4—C33—C32	3.5 (9)
O7^ii^—Zn2—N4—C29	−53.1 (6)	C29—N4—C33—C34	−177.2 (6)
O7^ii^—Zn2—N4—C33	132.3 (4)	C29—C30—C31—C2	−177.3 (7)
O7—C10—C12—C11	−99.1 (7)	C29—C30—C31—C32	2.2 (10)
O7—C10—C12—C13	85.1 (7)	C30—C31—C32—C33	−0.7 (10)
O8—C10—C12—C11	87.7 (8)	C31—C32—C33—N4	−2.2 (9)
O8—C10—C12—C13	−88.1 (7)	C31—C32—C33—C34	178.6 (5)
N1—Zn1—O1—C1	−7.1 (6)	C32—C33—C34—N3	177.4 (6)
N1—Zn1—N2—C22	4.2 (4)	C32—C33—C34—C35	−2.4 (9)
N1—Zn1—N2—C26	−178.4 (6)	C33—N4—C29—C30	−1.9 (10)
N1—C17—C18—C19	−0.3 (10)	C33—C34—C35—C36	−179.8 (5)
N1—C21—C22—N2	−1.2 (7)	C34—N3—C38—C37	−0.7 (11)
N1—C21—C22—C23	180.0 (6)	C34—C35—C36—C37	−0.2 (9)
N2—Zn1—O1—C1	71.5 (5)	C34—C35—C36—C39	−179.9 (6)
N2—Zn1—N1—C17	−179.5 (5)	C35—C36—C37—C38	−0.5 (10)
N2—Zn1—N1—C21	−4.9 (4)	C36—C37—C38—N3	1.0 (12)
N2—C22—C23—C24	0.5 (9)	C38—N3—C34—C33	−179.8 (6)
N3—Zn2—O5—C9	−54.4 (7)	C38—N3—C34—C35	0.0 (9)
N3—Zn2—N4—C29	178.3 (6)	C39—C36—C37—C38	179.2 (7)
N3—Zn2—N4—C33	3.6 (4)		

Symmetry codes: (i) *x*, *y*, *z*+1; (ii) *x*, *y*, *z*−1.

### Hydrogen-bond geometry (Å, º)

**Table:**

*D*—H···*A*	*D*—H	H···*A*	*D*···*A*	*D*—H···*A*
O1*W*—H1*WA*···O4^iii^	0.85	2.10	2.768 (6)	135
O1*W*—H1*WB*···O4^i^	0.85	1.79	2.644 (6)	179
O1*W*—H1*WB*···O3^i^	0.85	2.43	2.930 (5)	118
C17—H17···O4^iii^	0.93	2.36	3.202 (8)	150
C20—H20···O6^iv^	0.93	2.28	3.171 (8)	160
C23—H23···O6^iv^	0.93	2.40	3.306 (8)	164
C32—H32···O2^iii^	0.93	2.57	3.459 (8)	161
C35—H35···O2^iii^	0.93	2.45	3.353 (8)	165

Symmetry codes: (i) *x*, *y*, *z*+1; (iii) −*x*+1, −*y*+1, *z*+1/2; (iv) −*x*+1, −*y*+1, *z*−1/2.

## References

[bb1] Desiraju, G. R. (1996). *Acc. Chem. Res.* **29**, 441–449. 10.1021/ar950135n 23618410

[bb2] Desiraju, G. R. (2004). *Hydrogen Bonding in Encyclopedia of Supramolecular Chemistry*, edited by J. L. Atwood & J. W. Steed, pp. 658–665. New York: Marcel Dekker Inc.

[bb3] Dolomanov, O. V., Bourhis, L. J., Gildea, R. J., Howard, J. A. K. & Puschmann, H. (2009). *J. Appl. Cryst.* **42**, 339–341.

[bb4] Flack, H. D. (1983). *Acta Cryst.* A**39**, 876–881.

[bb5] Maspoch, D., Ruiz-Molina, D. & Veciana, J. (2007). *Chem. Soc. Rev.* **36**, 770–818. 10.1039/b501600m 17471401

[bb6] Nishio, M., Hirota, M. & Umezawa, Y. (1998). In *The C—H⋯π Interaction: Evidence, Nature and Consequences* Weinheim: Wiley-VCH.

[bb7] Ockwig, N. W., Delgado-Friedrichs, O., O’Keefee, M. & Yaghi, O. M. (2005). *Acc. Chem. Res.* **38**, 176–182. 10.1021/ar020022l 15766236

[bb8] Oxford Diffraction (2007). *CrysAlis PRO* Oxford Diffraction Ltd, Abingdon, England.

[bb9] Sheldrick, G. M. (2008). *Acta Cryst.* A**64**, 112–122. 10.1107/S0108767307043930 18156677

[bb10] Zang, S.-Q., Fan, Y.-J., Liang, R., Hou, H.-W. & Mak, T. C. W. (2011). *Cryst. Growth Des.* **11**, 3395–3405.

[bb11] Zang, S.-Q., Liang, R., Fan, Y.-J., Hou, H.-W. & Mak, T. C. W. (2010). *Dalton Trans.* **39**, 8022–8032. 10.1039/c0dt00374c 20657949

